# Multidrug-resistant *Acinetobacter pittii* is adapting to and exhibiting potential succession aboard the International Space Station

**DOI:** 10.1186/s40168-022-01358-0

**Published:** 2022-12-12

**Authors:** Braden T. Tierney, Nitin K. Singh, Anna C. Simpson, Andrea M. Hujer, Robert A. Bonomo, Christopher E. Mason, Kasthuri Venkateswaran

**Affiliations:** 1 Department of Physiology and Biophysics, Weill Cornell Medical College, New York, NY 10065 USA; 2 Jet Propulsion Laboratory, California Institute of Technology, Pasadena, CA 91109 USA; 3 Department of Medicine, Case Western Reserve University School of Medicine, Cleveland, OH 44106 USA; 4 Louis Stokes Cleveland Department of Veterans Affairs Medical Center, Cleveland, OH 44106 USA; 5 Departments of Biochemistry, Pharmacology, Molecular Biology and Microbiology, and Proteomics and Bioinformatics, Case Western Reserve University School of Medicine, Cleveland, OH 44106 USA; 6 CWRU-Cleveland VAMC Center for Antimicrobial Resistance and Epidemiology (Case VA CARES), Cleveland, OH 44106 USA

## Abstract

**Background:**

Monitoring the adaptation of microorganisms to the extreme environment of the International Space Station (ISS) is crucial to understanding microbial evolution and infection prevention. *Acinetobacter pittii* is an opportunistic nosocomial pathogen, primarily impacting immunocompromised patients, that was recently isolated from two missions aboard the ISS.

**Results:**

Here, we report how ISS-associated *A. pittii* (*n* = 20 genomes) has formed its own genetically and functionally discrete clade distinct from most Earth-bound isolates (*n* = 291 genomes). The antimicrobial susceptibility testing of ISS strains and two related clinical isolates demonstrated that ISS strains acquired more resistance, specifically with regard to expanded-spectrum cephalosporins, despite no prediction of increased resistance based on genomic analysis of resistance genes. By investigating 402 longitudinal environmental and host-associated ISS metagenomes, we observed that viable *A. pittii* is increasing in relative abundance and therefore potentially exhibiting succession, being identified in >2X more metagenomic samples in back-to-back missions. ISS strains additionally contain functions that enable them to survive in harsh environments, including the transcriptional regulator LexA. Via a genome-wide association study, we identified a high level of mutational burden in methionine sulfoxide reductase genes relative to the most closely related Earth strains.

**Conclusions:**

Overall, these results indicated a step forward in understanding how microorganisms might evolve and alter their antibiotic resistance phenotype in extreme, resource-limited, human-built environments.

Video Abstract

**Supplementary Information:**

The online version contains supplementary material available at 10.1186/s40168-022-01358-0.

## Introduction

Any bacterial species introduced into space will inevitably face the extreme pressures inherent therein (e.g., microgravity and solar radiation). While humans also encounter these challenges during space flight and, indeed, our physiology is impacted by prolonged exposure—microbes possess the unique ability to adapt quickly due to rapid cell division (thereby mutation) and the acquisition of external DNA elements through horizontal gene transfer (HGT) [1]. Given the pivotal roles microbes play in human health and physiology (e.g., ranging from pathogens to arbiters of a healthy gut), understanding how we can expect specific microbes to adapt to space-based ecosystems is essential for ensuring crew safety and health during longer space missions.

The International Space Station (ISS) is perhaps the paradigm of experimental “built environments” to observe such microbial adaptation in action. After all, all life on the ISS had to originate on Earth, and this makes it the ideal place to study, in a contained environment, how life changes in orbit. To date, there have been numerous other examples of microbes changing after being exposed to space. These include fungal adaptation to the ISS as a function of microgravity and changes in *Agrobacterium* genome composition, among others [2–4].

Recently, the microbe *Acinetobacter pittii* was isolated from surfaces within the ISS during two microbial-monitoring missions (referred to as microbial tracking-1 [MT1] and microbial tracking-2 [MT2]) [2, 5]. *A. pittii* is an opportunistic, Biosafety Level (BSL) 2 pathogen capable of causing potentially fatal infection, most commonly in immunocompromised patients in clinical settings [6, 7]. Bacteremia deriving from *A. pittii* has been observed as having a 17% 28-day mortality rate [8], though healthy individuals are usually not at risk of infection. Members of the genus *Acinetobacter* tend to be multi-drug-resistant and more commonly isolated from hospital settings as a result of their ability to resist environmental disinfectants and their potential for aerosolization [9]. In addition, *A. pittii* has been observed in various built environment settings other than hospitals (e.g., household objects) [10]. Many diverse *A. pittii* genomes are available from a variety of settings, making it an excellent model for comparative genomics.

The presence of a nosocomial BSL-2 pathogen in space warrants investigation in and of itself as the potential for human infection in this extreme environment raises concern; additionally, *A. pittii* is particularly adept at HGT in a simulated microgravity [1]. This indicates a propensity for rapid adaptation to conditions aboard the ISS and acquiring additional genes that could contribute to antibiotic resistance. As a result, monitoring the biology of *A. pittii*, and how strains are changing in space, is of high priority. Finally, understanding how an organism, especially a pathogen, is changing and whether it is propagating is critical to our ability to design effective infection prevention strategies. In other words, is it colonizing and spreading via the human body, or is it limited to the environment? Answering this metapangenomic [11] question requires both longitudinal sampling of human (e.g., the oral microbiome) and environmental (e.g., the space station viewports) metagenomes to identify the presence or absence of specific strains in complex microbial communities over time.

Here, we jointly used longitudinally collected isolates and metagenomic sequencing to measure changes to *A. pittii* genomes across time in space. We compared clinical strains isolated on Earth to cultured organisms from the space station and those de novo assembled into metagenome-assembled genomes (MAGs). We described the specific gene and phenotype changes possibly occurring as a function of exposure to space, and we identified where *A. pittii* is most prevalent across the ISS.

## Results

### *A. pittii* isolated from the ISS have become phylogenetically distinct from Earth-bound strains

We built a dataset comprising publicly available assembled genomes, metagenomic bins, and assembled, not-yet-public genomes that our team had sequenced from the ISS (Table 1). We downloaded 319 genomes annotated as *A. pittii* from the National Center for Biotechnology Information’s (NCBI’s) assembly database. Five of these were isolated from the ISS mission MT1 (flight 2), whereas 15 were from MT2 (flight 8) [12, 13]. The MT1/MT2 isolations were performed with similar swabbing and dilution protocols. We ran the genomes through GTDB-tK to confirm their taxonomy, removing eight from analysis due to their being annotated as other species (e.g., *Acinetobacter baumannii*).

**Table 1:** Characteristics of *A. pittii* genomes included in this study

**Genomes (*N*= 313,** ***A. pittii*)**			
**Source**	**Count**	**Source type**	**Reference**
MT1, flight 2	5	Environmental	Checinska Sielaff et al. 2016 [12]
MT1, environmental metagenomes	2	Environmental	Checinska Sielaff et al. 2016 [12]
MT2, flight 8	15	Environmental	Simpson et al. 2021 [13]
Non-human/environmental	29	Other	https://www.ncbi.nlm.nih.gov/genome/browse#!/prokaryotes/2516/
Human	199	Host-associated	https://www.ncbi.nlm.nih.gov/genome/browse#!/prokaryotes/2516/
Unknown	63	Other	https://www.ncbi.nlm.nih.gov/genome/browse#!/prokaryotes/2516/
**Metagenomes (*N*= 402)**			
**Source**	**Count**	**Source type**	**Reference**
MT1 environmental swabs	42	Environmental	https://www.ncbi.nlm.nih.gov/sra/?term=PRJNA438545)
MT2 environmental swabs	60	Environmental	https://genelab-data.ndc.nasa.gov/genelab/accession/GLDS-252/
MT2 crew, ear	30	Host-associated	Morrison et al. 2021 [5]
MT2 crew, mouth	31	Host-associated	Morrison et al. 2021 [5]
MT2 crew, nasal	30	Host-associated	Morrison et al. 2021 [5]
MT2 crew, pooled samples	31	Host-associated	Morrison et al. 2021 [5]
MT2 crew, saliva	124	Host-associated	Morrison et al. 2021 [5]
NASA Twins Study, Gut	22	Host-associated	Garrett-Bakelman et al. 2019 [14]
NASA Twins Study, Saliva	32	Host-associated	Garrett-Bakelman et al. 2019 [14]

Additionally, in a dataset of 402 short-read metagenomes from ISS crew and environmental sites on the ISS, we assembled metagenome-assembled genomes (MAGs) in the hope of finding additional *A. pittii* strains. These samples comprised 42 from environmental sites on the MT1 mission, 60 from environmental sites on the MT2 mission, 246 from different crew body sites on the MT2 mission, and 54 from the crew of the NASA Twins Study, which took place prior to the MT1/MT2 missions [5, 14]. We annotated only two MAGs as *A. pittii* using GTDB-tK. Both genomes were >99% complete, had <1% contamination, and were from environmental samples from the MT1 mission (samples SRR6853346 and SRR6853362, both from flight 2). These brought the total number of *A. pittii* genomes (including MAGs and isolates) carried forward for the rest of the analysis to 313 (Table 1, Supp Table 1).

**Fig. 1:**
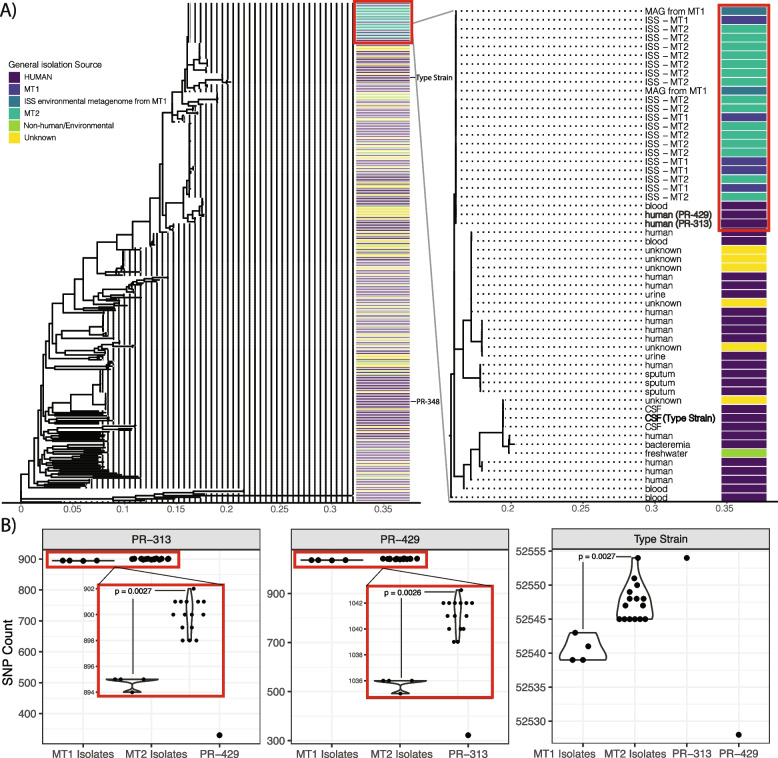
*A. pittii* from the ISS as compared to publicly available genomes. **A** A phylogenetic tree displaying the “ISS clade” (red box), which contains all ISS genomes as well as 3 Earth-derived strains. The right panel is a zoomed-in image on the upper portion of the tree. **B** SNP level similarity of the ISS isolates and two closely related Earth strains as well as a related patient isolate, referred to as “type strain.” Names in the gray bar are the identity of the reference genome to which all others on the *x*-axes were compared. *P* values were generated from Wilcox tests. CSF refers to cerebrospinal fluid

We then built phylogenetic trees (with four different approaches ranging from marker-gene-based to SNP-based) for the resultant strains of *A. pittii* isolated from or identified on the ISS versus those from different Earth environments (Fig. 1, Supp Figs. 1 and 2). We identified that ISS *A. pittii* isolates formed a distinct clade from strains associated with all other ecosystems. Across all trees, only two strains, both isolated from infected patients, from Earth additionally fell in this clade. Given their placement, we focused specifically on grounding our proximal functional, antimicrobial, and genetic analysis of the ISS strains in comparison to these two strains (referred to as PR-313 and PR-429). By comparison, isolates from other ecosystems (e.g., human blood, wastewater) did not cluster together based on phylogeny to the same level as those from the ISS, and, overall, other *A. pittii* from the environmental ecosystems were phylogenetically distinct from the ISS strains.

We additionally compared PR-313, PR-429, and the assembly of the *A. pittii* type strain (GenBank accession GCA_000369045.1, ATCC 19004, acquired from a patient blood sample) to the ISS isolates (not including the MAGs) at the SNP-level (Fig. 1B). ISS *A. pittii* strains differed from the type strain by an average of 52,546 SNPs. They differed from PR-313 and PR-429 by 899 and 1040 SNPs on average, respectively. The MT2 isolates had more SNPs relative to all three references than the MT1 isolates (*p* < 0.001).

### *A. pittii’s* potential succession through the built environment of the ISS

Next, we aimed to determine the source and observe the potential succession of *A. pittii* on the ISS based on shifts in relative abundance (as opposed to absolute abundance), which is an approach we have taken in prior work [15]. In the same shotgun sequenced samples from which we identified our metagenomic bins, we computed the abundance of *A. pittii* using Kraken2. We stratified this analysis by metagenomes that had, versus had not, been treated with propidium monoazide (PMA) to remove non-viable cells.

**Fig. 2:**
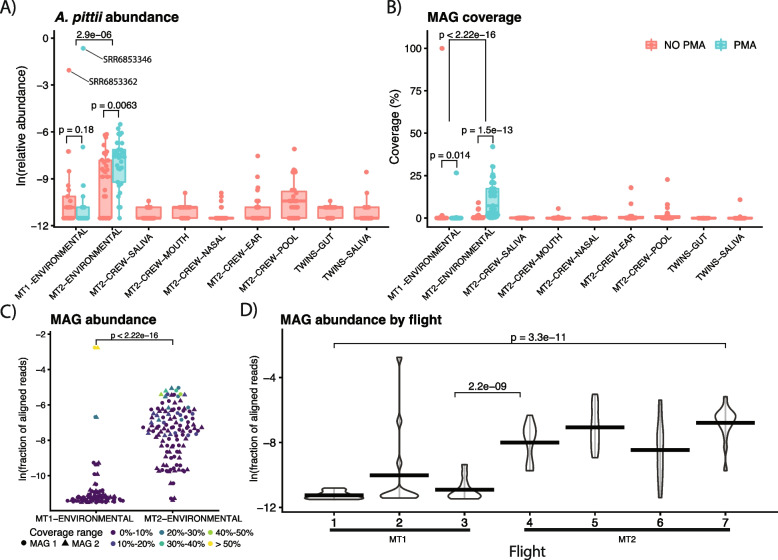
The distribution and potential succession of *A. pittii* aboard the ISS. All *p* values are derived from Wilcox tests. **A** The relative abundance of *A. pittii* across different metagenomes from human subjects as well as the ISS environment. The *x*-axis indicates the source of the metagenomes, with “pool” referring to combined samples across sites. **B** The percent genome coverage with at least 3 reads aligning to each of the two *A. pittii* metagenome-assembled genomes (MAGs) identified in our analysis. **C** The proportion of reads aligning to each of these MAGs from environmental metagenomes. **D** The abundance of *A. pittii* strains as a function of specific flights. The proportion of reads aligning to MAGs broken down by each flight in the MT1 and MT2 missions. *P* values in this panel correspond to the change in abundance between the first and last flights (top) and the different missions (bottom)

We noted the potential succession of viable *A. pittii* throughout the environment of the ISS (Fig. 2A). We identified more reads mapping to *A. pittii* in the environmental samples than in the human samples, and its relative abundance was higher in PMA-treated environmental metagenomes (*p* = 0.0063) in the MT2 mission. Overall, its abundance increased between the two missions (*p* < 0.001). The majority of *A. pittii* abundance during the MT1 flight was in a small number of samples. One environmental metagenome from the MT1 mission had extremely high levels of *A. pittii* abundance (SRR6853346, relative abundance = 52.1%). This and the MT1 sample with the second-highest abundance (SRR6853362, relative abundance = 12.8%) were the ones we were able to assemble MAGs from, and they were taken from the port panel next to Cupola node 3 on the ISS. The median relative abundance of *A. pittii* (0.02%) during the MT2 mission was the highest by an order of magnitude compared to the other metagenomic sources. Further, short reads mapped to *A. pittii* with Kraken2 were identified in more locations across the ISS in MT2 than in MT1 (19/42 [45.2%] metagenomes in MT1 vs. 48/60 [80%] in MT2).

To further explore the spread of *A. pittii* throughout the ISS, we next searched specifically for the presence of the two MAGs we identified previously in all the environmental metagenomic samples in our dataset. We computed the fraction of raw reads in each metagenome aligning back to the MAGs (Fig. 2B, C). We observed a statistically significant (*p* < 0.001) increase in both the coverage and relative abundance of the genomes. Metagenomes treated with PMA had higher coverage (*p* < 0.05) in both missions.

To gain an even higher-resolution view into the potential succession of *A. pittii* aboard the ISS, we broke down its shifts in abundance as a function of flight. The MT1 mission consisted of three longitudinal flights (i.e., time points), whereas the MT2 consisted of four, with increasing average abundance from flight one to seven in *A. pittii* MAGs (Fig. 2D). Changes in abundance between flights 1 and 7 were statistically significant (*p* < 0.001), as well as between flights 3 and 4 (the gap between the missions).

### ISS-associated *A. pittii* are becoming functionally distinct from Earth-bound strains

We next aimed to determine how the ISS *A. pittii* varied genetically and functionally compared to its Earth counterparts. We identified and functionally annotated all the Open-Reading-Frames (ORFs, which refer to the coding sequences of a microbial genome) in each of the 313 genomes/MAGs. In total, we identified 1,188,894 genes and 1459 distinct Clusters of Orthologous Gene (COG) annotations (Supp Table 2).

Initially, we sought to identify similarities and differences in COGs annotated to ORFs present in five different genome “types”: the genomes of the MT1 and MT2 isolates, the MAGs, the type strain, and the two human isolates that were consistently grouped in the ISS clade (as described in Fig. 1). Nineteen COGs were present in all genome types except the type strain. These included two transketolases, a type II secretion mechanism (PulG), a sulfoxide reductase and sulfatase maturation enzyme, an alcohol dehydrogenase, a curcumin reductase, a Zn-dependent reductase (and a Zn2+ transport system), and an integrase, among others. Notably, every ISS-isolate uniquely contained the transcriptional repressor LexA, which was not shared with the type strain or otherwise similar genomes.

In our genome collection, we also searched against the CARD database to identify antimicrobial resistance (AMR) genes (Supp Table 2). In addition, for ISS and clinical strains used to assess antimicrobial phenotypic characteristics, we searched genomes against the CARD, NCBI, ARG-ANNOT, and ResFinder databases and verified predicted AMR status and gene identity of flagged sequences (those not having a 100% identity match) by blasting against GenBank’s non-redundant protein sequence database. We identified a bolus of AMR markers, with the 20 ISS genomes possessing a total of 10 distinct resistance gene families (9/10 gene families were in every ISS genome). Each genome contained potential resistance to 11 drug classes through 4 general resistance mechanisms (antibiotic efflux, antibiotic inactivation, target alteration, and reduced permeability for the antibiotic). However, the presence of resistance mechanisms and genes did not discriminate between the Earth and ISS-associated strains. However, the ISS-genomes did hierarchically cluster primarily with human-associated strains and contained a large diversity of genes that could potentially confer antibiotic resistance (Supp Fig. 1).

### Antimicrobial phenotypic characteristics of *A. pittii* strains

**Fig. 3:**
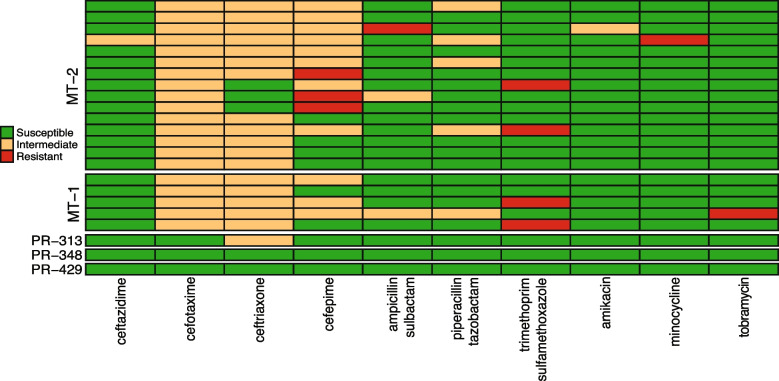
Antimicrobial susceptibility testing data of the various *A. pittii* strains. The results of antimicrobial testing of the ISS isolates vs. comparable clinical isolates. See Supp Table 2 for a more detailed view of these analyses

We tested 16 clinically relevant antibiotics for which the Clinical and Laboratory Standard Institute (CLSI) has established breakpoints for *Acinetobacter* species, as these are the commonly used antimicrobial agents administered to treat infections caused by this opportunistic pathogen (Fig. 3, Supp Table 2). The well-characterized Earth strains PR-313, PR-348, and PR-429 isolated from clinical samples were used as comparators in this study [16]. Clinical isolates PR-313 and PR-429 were the two strains that consistently grouped phylogenetically with the ISS strains in this study (Fig. 1A). PR-429 was placed distantly in our phylogenetic analysis. These three strains were susceptible to all antibiotic compounds tested, with the exception that PR-313 showed resistance to ceftriaxone (MIC = 32 μg/ml, intermediate resistance, I) as did ISS strains.

All ISS *A. pittii* isolates (*n* = 20) tested were susceptible to the commercially available and often used carbapenems: meropenem and imipenem; the fluoroquinolones: ciprofloxacin and levofloxacin; the aminoglycoside gentamicin; and tetracycline. The susceptibility to the other antimicrobials is typical of *A. pittii*. The isolates were also predominantly susceptible to minocycline [19/20 isolates; one isolate MIC > 8 μg/ml, resistant (R)], and the aminoglycosides amikacin (19/20 isolates; one isolate MIC = 32 μg/ml, I) and tobramycin (19/20 isolates; one isolate MIC > 8 μg/ml, R). Susceptibility to ceftazidime was more notable, as only one ISS isolate demonstrated resistance (MIC = 16 μg/ml, I). The potency of the cephalosporins varied widely as can be seen from the MICs listed in Supp Table 2.

All ISS isolates demonstrated resistance to cefotaxime (MICs 16–32 μg/ml, I), and unlike the clinical isolates (*n*=3), most ISS strains were also resistant to cefepime (14/20 isolates: MIC = 16 μg/ml, *n* = 11, I; MIC > 16 μg/ml, *n* = 3, R) and ceftriaxone (17/20 isolates: MIC = 32 μg/ml, I). For β-lactam/β-lactamase inhibitor combinations, 3/20 isolates were resistant to ampicillin-sulbactam (MIC = 16/8 μg/ml, *n* = 2, I; MIC > 16/8 μg/ml, *n* = 1, R), and 5/20 isolates were non-susceptible to piperacillin-tazobactam (MIC = 32/4 μg/ml, I). In addition, 4/20 isolates were resistant to a combination of trimethoprim-sulfamethoxazole (MIC > 2/38 μg/ml, R). One strain each from MT-1 (IIF1SW-P4) and MT-2 (F8_7S_12B) flight mission exhibited multi-drug resistance (6 out of 16 antimicrobials) and were isolated from Cupola (IIF1SW-P4) and US Lab (F8_7S_12B) locations.

### ISS-bound *A. pittii* contain genetically discrete accessory genomes from Earth-bound strains

**Fig. 4:**
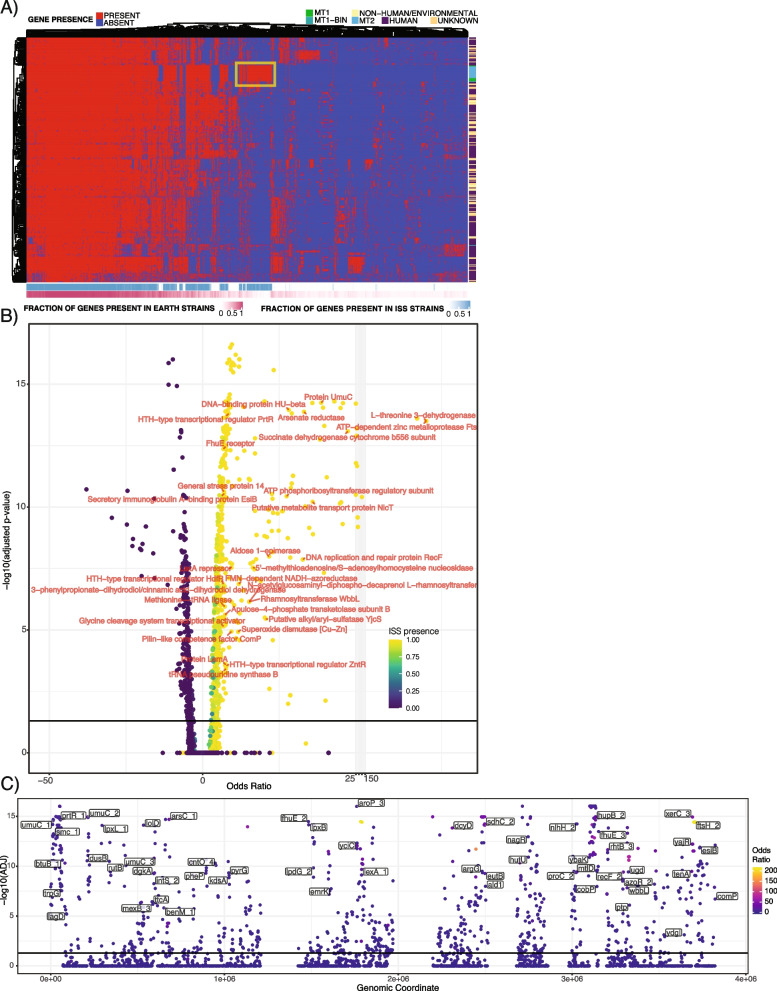
Identifying the ISS-associated-accessory genes of *A. pittii*. **A** A hierarchically clustered visualization of the *A. pittii* pangenome. Each column is a gene, and each row is a sample. The bottom rows indicate the proportions of ISS or Earth isolates in which a given gene was identified. The yellow box indicates the presence of genes particularly associated with the genomes of ISS strains. **B**, **C** The output of a gene-level association study comparing ISS and Earth strains. The volcano plot in **B** indicates the distribution of odds ratios between genes enriched in and lost in space flight. The top 30 most ISS-associated, non-hypothetical, distinct proteins with the highest odds ratios are labeled. Point color corresponds to the fraction of ISS genomes in which a gene is found. **C** The genomic context of significant, non-hypothetical, genes. The *x*-axis is the concatenation of all contigs in a representative ISS isolate. Blank spots on this axis contain genes or portions of the genome that were not evaluated in the association study due to the low prevalence

Our prior analyses provided a sense of functions that were gained or lost by different strains of *A. pittii*; however, they do not necessarily capture difficult-to-annotate sequences (i.e., genetic dark matter) or provide a sense of gene-level (or mutational-level) similarity between isolates. To explore this space, we identified a total of 43,948 core and accessory genes across all *A. pittii* isolates and MAGs. Via hierarchically clustering the consensus genes from the pangenome, we observed a set of sequences that were present in 100% of ISS genomes and only a small proportion (< 10%) of Earth genomes (Fig. 4A, yellow box).

This observation motivated us to carry out a gene-level, pangenome association study with the aim of determining the “ISS-associated-accessory genome” of *A. pittii*. Adjusting for population stratification due to shared lineage, we used logistic regression to test the association between 7411 gene sequences that were found in between 5 and 100% of all samples. After correcting for the false discovery rate, we identified a total of 175 significant positive associations with the ISS that were present in 100% of ISS *A. pittii* and <10% of the Earth strains (Fig. 4B, Supp Table 3). These genes had limited annotations, occurred across the genome (Fig. 4C), and corresponded to 15 unique, non-hypothetical proteins that included UmuC (involved in ultraviolet radiation protection), DNA repair (e.g., RecF), the FhuE receptor for ferric iron uptake, arsenate reductase, and other stress-response proteins (e.g., the ATP-dependent zinc metalloprotease FtsH).

However, a gene-level association study does not necessarily identify the truly unique mutational signatures of space-bound *A. pittii*. This analysis needs to be grounded in a clear comparison to the most closely related Earth-bound strains. With this in mind, we computed a single-nucleotide polymorphism (SNP) and insertion/deletion (indel) genome-wide association study (GWAS, Supp Table 3) using the strains in the right panel of Fig. 1A, which were most closely related to the ISS strains. We used PR-313 as the reference genome for joint variant calling across the 40 genomes for which we were able to find raw reads.

**Fig. 5:**
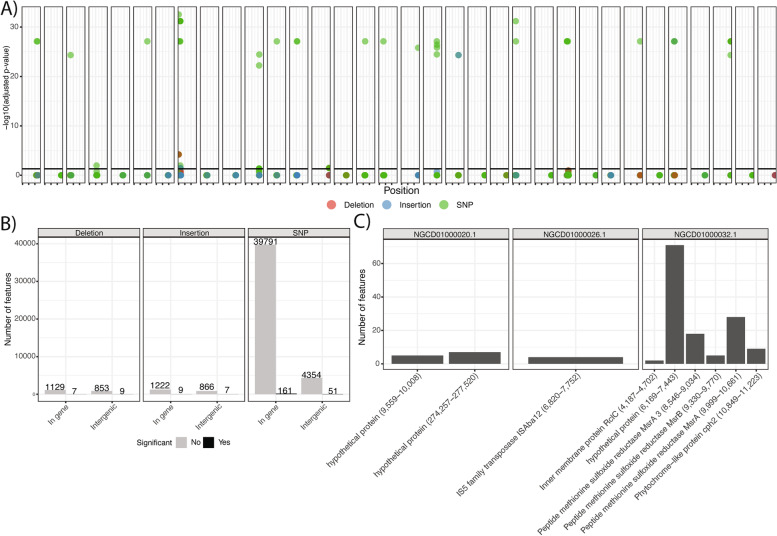
The SNPs and indels associated with ISS *A. pittii*. **A** The position on different contigs of statistically significant, ISS-associated, mutational changes, and the *y*-axis is adjusted *p* value. The solid line is an adjusted significance cutoff of 0.05. **B** A summary of the results for our GWAS in terms of the number of significant findings versus the total number of features identified in joint variant calling. **C** Open-Reading-Frames (genes) with the top number of associated features. Numbers on the *x*-axis correspond to a position on a given contig

Joint variant calling yielded >40,000 variations across all 40 genomes. The majority of significantly associated features with the ISS were SNPs, and they occurred across all contigs (Fig. 5A). Fifty-one of these SNPs were ostensibly outside of coding sequences, whereas only 161 were in open Open-Reading-Frames (Fig. 5B). A total of 16 insertions and 16 deletions, half of which were intergenic, were statistically associated with spaceflight.

Despite many associated features scattered around the genome, there were a few key genes and regions that seemed to have more mutational burden than others. In other words, these portions of the genome had the greatest variation relative to the most closely related Earth-bound strains. The genes with more than one mutation are reported in Fig. 5C with their relative position on different contigs. Three of these coding sequences were consecutive regions in the genome and coded for three different methionine sulfoxide reductases, which are involved in oxidative stress response [15]. The gene with the most protein variation was hypothetical and had no known annotation, even when annotated by alignment to the UniRef90 database.

## Discussion

Here, we report the progressive changes in the genetics and abundance of *A. pittii* aboard the ISS. We demonstrated that it forms a genetically and functionally distinct clade set apart from most genomes isolated on Earth and that the ISS strains appear to be prevalent and changing in relative abundance through the environment of the space station. We note that none of the ISS isolates or Earth-based isolates were isolated at or stored at the same facility, ruling out contamination as a potential source of genomic similarity between the closely-related strains. In addition to characterizing functions that *A. pittii* is gaining on the ISS, we additionally determined a set of 175 genes and 208 SNPs/indels enriched in ISS-associated genomes.

Our study, of course, is not without drawbacks. First, we cannot prove true succession without ecological-scale analysis or increasing absolute abundance without a measure such as qPCR. As a result, we only argue for “potential succession.” We feel this hypothesis is strengthened given the fact that with consistent swabbing and culturing approaches, 3X more colonies were identified across the MT2 flights compared to MT1. Second, in our analysis of coverage in Fig. 2B, the higher values we observed ranged from 25 to 50%—while the increase between missions is encouraging, deeper sequencing would help to further confirm the continued presence of *A. pittii* and recover additional MAGs.

While *A. pittii* is primarily a risk to immunocompromised individuals (who have historically not been astronauts), the proliferation of private spaceflight has enabled greater access to space for individuals of diverse medical backgrounds. Consider the recent Inspiration4 mission, where a cancer survivor and amputee was one of four crew members. As a result, comparative genomics on even low-risk pathogens, especially those with a propensity for HGT, is worth reporting.

The functions and genes gained and lost by *A. pittii* over the course of the MT1 and MT2 missions are particularly interesting. These results, of course, could be strengthened by checking gene annotations with an even wider array of software. The fact that 100% of MT2 strains had the RelBE toxin-antitoxin system may indicate an increased ability to withstand osmotic stress (Supp Table 2) [17]. Similarly, an increased number of transposases and transcriptional regulators could potentially improve fitness in the harsh environment of the ISS. Conversely, the gain of genes and functions associated with sulfate and metal oxidation (Fig. 4) could indicate *A. pittii* swapping to alternative carbon sources more suited to the ISS.

LexA was the only COG found uniquely across all ISS-associated strains and not identified in any Earth strains. We hypothesize that this indicates possible selection pressure on *A. pittii*’s DNA damage response, due to high radiation levels and other harsh conditions aboard the ISS. The LexA protein is a key regulator of the SOS response to DNA damage in *Escherichia coli* and many other bacteria [18–20]. The protein RecA (which was flagged in our gene-level association study as well), when bound to ssDNA, cleaves LexA. This de-represses transcription of multiple SOS genes, which halt cell division and lysis, initiate DNA repair, and induce mutagenesis directed by error-prone polymerases, as well as partially regulating toxin-antitoxin activity and biofilm formation [20, 21]. *Acinetobacter* species normally lack the LexA protein and have a separate mechanism for SOS signaling and response to stress that requires RecA, which is not well-understood [22]. Multiple genes, including *umuDAb* and *ddrR*, which are unique to *Acinetobacter* appear to serve a similar role to *lexA*, but only for a subset of SOS genes; research continues in this area since antibiotic resistance in *Acinetobacter* as a result of SOS mutagenesis is a pressing problem for human health [23, 24]. It is potentially concerning that *A. pittii* ISS strains have acquired a part of the SOS pathway that related *Acinetobacter* isolates lack. Whether *lexA* detected in all ISS strains of *A. pittii* is being expressed and whether acquired LexA could actually serve as a repressor of SOS response genes in *Acinetobacter* species, would require significant further study as this has potential implications for the emergence of resistance to a variety of antibiotics. One possibility is that an active acquired LexA suppressor might give a competitive growth advantage for *A. pittii* in the space environment by slowing the SOS response, which would be highly active due to DNA damage from the high-radiation environment of the ISS.

The observation of three back-to-back proteins with a large mutational burden, all being methionine sulfoxide reductases, is particularly striking. Compared to other amino acids, methionine residues are more likely to undergo oxidation. Peptide methionine sulfoxide reductase (MSR) enzymes appear to protect cells from widespread oxidative damage by restoring function to proteins with oxidized methionine residues, though in some species such as *S. aureus*, these repair enzymes are expressed in response to antibiotics or sunlight exposure rather than oxidation [15]. The enzymes MsrA and MsrB repair oxidative damage to the (S) and (R) forms of methionine sulfoxide, respectively [25]. In bacteria, certain deletions in *msrA* and *msrB* can lead to species-specific effects such as decreased resistance to oxidants and cleaning products, decreased antibiotic resistance, decreased virulence, and decreased ability to survive within a eukaryotic host [26] For example, *Salmonella typhimurium msrA* knockout mutants are 3000x more sensitive to hypochlorite and less able to survive inside a host cell [3, 27]. However, we do not know how specific mutations in the *msrA* and *msrB* genes of the ISS *A. pittii* affect its functionality.

The antimicrobial resistance results are also of interest, especially given that while carrying similar genetic signatures for antimicrobial resistance, ISS *A. pittii* have a discrete phenotypic profile from similar Earth strains. The susceptibility to meropenem and imipenem is consistent with the genetic analyses that did not reveal the common carbapenemases in *Acinetobacter* species that confer a carbapenem-resistant phenotype (no detection of the major groups *bla*_OXA-23-like_, *bla*_OXA-24/40-like_, *bla*_OXA-235-like_, *bla*_NDM_, *bla*_VIM_, or *bla*_IMP_). Cefepime and ceftazidime are often used as a therapy against *Acinetobacter* infections when susceptibility is detected, but resistance to cefepime in ISS strains might be related to the microgravity stress. The intrinsic *Acinetobacter-*derived cephalosporinase *bla*_ADC-18_ and the intrinsic oxacillinase *bla*_OXA-500_ genes were detected in all isolates except for outgroup strain PR-348, in which the cephalosporinase *bla*_ADC-150_ and the oxacillinase *bla*_OXA-421_ were detected. In addition, all isolates appeared to be clonal and were Pasteur multi-locus sequence type 64 [28]. Strains of *A. pittii* isolated aboard the ISS on two different missions separated by nearly 4 years appear to all belong to the same clonal lineage; only two Earth strains collected, sequenced, and deposited into GenBank thus far, from human patients, belong to this same lineage.

The resistance of some ISS strains to ampicillin/sulbactam is particularly significant, as this β-lactam/β-lactamase inhibitor combination is often used in the treatment of multidrug-resistant *Acinetobacter* species infections [29–31]. Antimicrobial resistance exhibited by the ISS isolates tested in this study specifically with regard to cefotaxime and cefepime was notable since the two related strains isolated from patients were not resistant. The enhanced AMR phenotype by the ISS strains is interesting, and this phenomenon should be confirmed by exposing the susceptible Earth strains to simulated microgravity conditions to understand whether microgravity influences the enhanced AMR in *Acinetobacter* species.

## Conclusions

A microbe’s ability to grow and survive in variable conditions is defined by its ability to respond to various environmental demands under suboptimal conditions. The advantage of microbes, having a short generation time, is that their mutational frequency allows for great genetic diversity. Here, we describe the “ISS effect,” where microbes may adapt to the extreme environment of the ISS through mechanisms that enhance their survival and may contribute to augmented antimicrobial resistance (despite not being exposed to antimicrobials). We show the potential ecological succession of *A. pittii* aboard the ISS as well as the longitudinal effects of the ISS effect via increased mutational frequencies and acquisition of further stress-associated protein-coding genes. We additionally identify particular, highly variable, sequences of interest (i.e., MSR enzymes) that are worth further investigation as spaceflight-impacted genes. Overall, we found that the extreme environment of the ISS is possibly exacting an intense selective pressure on *A. pittii*, and the nature of the genomic changes warrant continued observation in the years to come.

## Methods

### Downloading and annotating public *A. pittii* genomes

Using the NCBI’s entrez command line, we downloaded every genome annotated as *A. pittii* (NCBI taxonomic ID48296) from the NCBI’s assembly database. These downloaded genomes included those from the MT1 and MT2 missions as well as others isolated from the ISS. We manually searched the NCBI BioProject, BioSample, and assembly databases for all 319 downloaded *A. pittii* genomes in order to determine their environment. We manually mapped environmental tables to a consistent set of terms (e.g., converting “endobronchial tube” to “respiratory tract” and searching BioSample for information on isolation sources stored in a non-standard format). The original terms, as well as the mapping therein, is reported in Supplementary Table 1.

### Phylogenetic annotation and computing SNP distances

To confirm the taxonomy of all assembled genomes as well as those downloaded from the NCBI, we used GTDB-Tk (V1.6.0), the Genome Taxonomy Database’s accompanying toolkit that uses a variety of dependencies to place genomes into a phylogenetic tree according to a set of globally conserved marker genes [32–38]. Specifically, we sequentially employed GTDB-Tk’s identify, align, and infer functions, all with the default settings in order to taxonomically annotate and build a phylogenetic tree from our downloaded and assembled genomes. We additionally used GTDB-Tk’s resultant taxonomic classification to filter out any NCBI genomes that were not annotated as *A. pittii.* For Supp Fig. 2, we plotted and annotated the GTDB-Tk phylogenetic tree in R (V4.1.1) using ggtree (V 3.0.4) [39].

For the pairwise SNP distance comparisons in Fig. 1B, we identified a core-SNP set using snippy V4.6.0 (https://github.com/tseemann/snippy) for the ISS clade (i.e., all ISS MAGs/genomes and the two comparator Earth-based patient isolates we referenced throughout the study). We ran Snippy with the default settings, and its output was also used with gubbins V3.2.1 [40] and FastTree V2.1.11 [41] (also with default settings as recommended on Snippy’s README) to generate the phylogenetic tree in Supp Fig. 1B.

### Pangenome construction, functional annotation, and joint variant calling

We first identified ORFs with Prokka V1.14.6 [42] using the default parameters. The ab initio functional annotations (i.e., COGs) for each predicted gene sequence were identified as part of Prokka’s automated pipeline, which pulls annotations from a variety of reference databases. To build a pangenome, we used ROARY running the default settings on the Prokka output. We called SNPs and indels jointly across the closely related Earth/ISS strains using SPANDx (V4.0.1) [43] with the default settings.

### Alternate phylogeny construction

We additionally computed phylogenies for Supp Fig. 1A and Fig. 1A using the output of SPANDx (the maximum likelihood tree) and RAXmL (V8.2.12, with the following parameters: -m GTRGAMMA -p 12345), which we ran on the ROARY core alignment output.

### Association studies

We used the presence-absence matrix output of ROARY for the gene-level association study and the filtered VCF output of SPANDx for the SNP/indel association study. We computed associations with pyseer (V1.3.9) [44]. For the gene-level analysis, we adjusted for population stratification with five principal components based on phylogenetic distances generated from the tree in Fig. 1. These distances were computed with pyseer’s built-in scripts. For the mutational analysis, we used pyseer’s mixed modeling approach with similarities built from SPANDx’s maximum likelihood phylogenetic tree.

### Accessing metagenomic datasets

We downloaded publicly available environmental metagenomic datasets from the MT1 (accession = PRJNA438545), MT2 (accession = PRJNA781277) missions, and genomes of 402 *A. pittii* isolates of human clinical samples. The microbial sequencing associated with astronaut samples of MT-2 study and NASA Twins study cannot be shared publicly due to Institutional Review Board (IRB). These data are available upon request in NASA Life Sciences Data Archive (LSDA) (https://lsda.jsc.nasa.gov/Dataset).

### Processing and de novo assembly of ISS metagenomic data

We first ran a quality control pipeline on all metagenomic samples. We used bbtools (V38.92) functions to clump reads, remove adapter contamination, and remove sequencing error (clumpify [parameters: optical=f, dupesubs=2,dedupe=t], bbduk [parameters: qout=33 trd=t hdist=1 k=27 ktrim="r" mink=8 overwrite=true trimq=10 qtrim='rl' threads=10 minlength=51 maxns=-1 minbasefrequency=0.05 ecco=f], and tadpole [parameters: mode=correct, ecc=t, ecco=t]) [45]. Where necessary, we would remove unmatching reads using bbtool’s repair function (default parameters). We additionally used bowtie2 (parameters: --very-sensitive-local) to align to the human reference genome (GRCh38) [46]. We de novo assembled all metagenomic reads into contigs using metaSPAdes [47] (default settings).

### Constructing metagenome-assembled genomes (MAGs)

We constructed MAGs from all metagenomic samples using an approach modeled after the literature [48]. We used bbmap [45] to align quality-controlled reads from each sample to its respective contigs resulting from the assembly. We used the alignment output with the default settings, as well as the contigs themselves, as input into MetaWRAP V1.3 (which calls metaBAT2, Maxbin2, and CONCOCT) to generate MAGs [49–52]. For each sample, we used dRep V3.2.2 [53] (parameters: -p 15 -comp 50 -pa 0.9 -sa 0.95 -nc 0.30 -cm larger) to estimate the quality of each genome and to remove redundant genome bins. We did not dereplicate genomes across samples to avoid combining distinct *A. pittii* strains. The quality of both *A. pittii* bins can be found in Supp Table 1. We classified the taxonomy of each within-sample non-redundant bin with GTDB-Tk in the same manner as for the isolate genomes.

### Analysis of CARD genes within isolate and bin genomes

We used RGI (main) V5.2.1 running the default settings to identify antibiotic resistance genes and markers in the isolate genomes and metagenomic bins.

### Identifying *A. pittii* in metagenomic samples

We used Kraken2 and Bracken (running the default settings) to estimate the abundance of *A. pittii* in each of our quality-controlled metagenomes [54, 55]. To search for the abundance of the specific MT1/MT2 cultured isolates and the metagenome-assembled genomes, we aligned the raw reads of each metagenome against the contigs for a given genome. We counted the number of reads aligning to a genome and, to adjust for depth of sequencing, divided by the number of read mate-pairs in each sample. As a point of comparison, we additionally included the high-quality, assembled genome of recently isolated *A. pittii* type (NCBI assembly ID = GCA_000369045, https://www.ncbi.nlm.nih.gov/biosample/SAMN01828154/), referred to as a type strain in this analysis.

### Antimicrobial susceptibility testing (AST)

Susceptibility testing using standard antibiotics was performed by broth microdilution with MicroScan Gram-negative NM56 trays (Beckman Coulter Inc., Brea, CA). *Pseudomonas aeruginosa* and *Escherichia coli* ATCC 25922 were used as control strains as recommended in CLSI methods. Minimum inhibitory concentrations (MICs) were interpreted according to the 2021 Clinical and Laboratory Standards Institute (CLSI) guidelines for *Acinetobacter* species [56].

## Supplementary Information

**Additional file 1: Supplementary Table 1.** Summary statistics for *A. pittii* genomes, metagenomic assemblies, and Metagenome-Assembled-Genomes.

**Additional file 2: Supplementary Table 2.** Summary of CARD (i.e., *in silico*) and phenotypic antimicrobial resistance data in the ISS vs. the Earth genomes. Also contains summary of COG annotations per genome.

**Additional file 3: Supplementary Table 3.** Output of associations described in Figs. 4 and 5.

**Additional file 4: Supplementary Figure 1.** Two alternate phylogenetic trees built with different SNP-calling pipelines, A) SPANDx and B) Snippy. The former contains the genomes most closely related to the ISS *A. pittii* isolates for which we were able to access raw reads. The latter was generated with raw reads for the ISS isolates and the two closely related Earth strains that were used in antibiotic testing.

**Additional file 5: Supplementary Figure 2.** A cladogram from GTDB-tK taxonomic annotations comparing the A. pittii strains from earth versus those identified on the International Space Station. We label the clade containing ISS-associated microbes as the “ISS clade”. We additionally indicate the position of the three clinical isolates that underwent antimicrobial resistance screening as reported in Supplementary Table 2. The labeled “blood strain” lies in the ISS clade in Fig. 1.

**Additional file 6: Supplementary Figure 3.** The presence of Antimicrobial Resistance Ontologies (AROs) across all genomes.

## Data Availability

The MT1 (accession = PRJNA438545), MT2 (accession = PRJNA781277) datasets, and genomes of 402 *A. pittii* isolates of human clinical samples are available at NCBI (https://www.ncbi.nlm.nih.gov/datasets/genomes/?taxon=48296). The microbial sequencing associated with astronaut samples of MT-2 study and NASA Twins study cannot be shared publicly due to IRB. These data are available upon request in NASA Life Sciences Data Archive (LSDA) (https://lsda.jsc.nasa.gov/Dataset). Processed data from this study, including the isolate gene catalogs and Kraken2/Bracken abundances, are available at https://figshare.com/projects/Multidrug-resistant_Acinetobacter_pittii_is_adapting_to_and_exhibiting_succession_aboard_the_International_Space_Station/133467. All code used in this project is documented and available at https://github.com/b-tierney/space_genomes.
