# Supplementary figures and images for: Multidrug-resistant Acinetobacter pittii is adapting to and exhibiting potential succession aboard the International Space Station
Source: Microbiome. 2022 Dec 12;10:210. doi: 10.1186/s40168-022-01358-0 (PMC9743659; doi:10.1186/s40168-022-01358-0)

A)

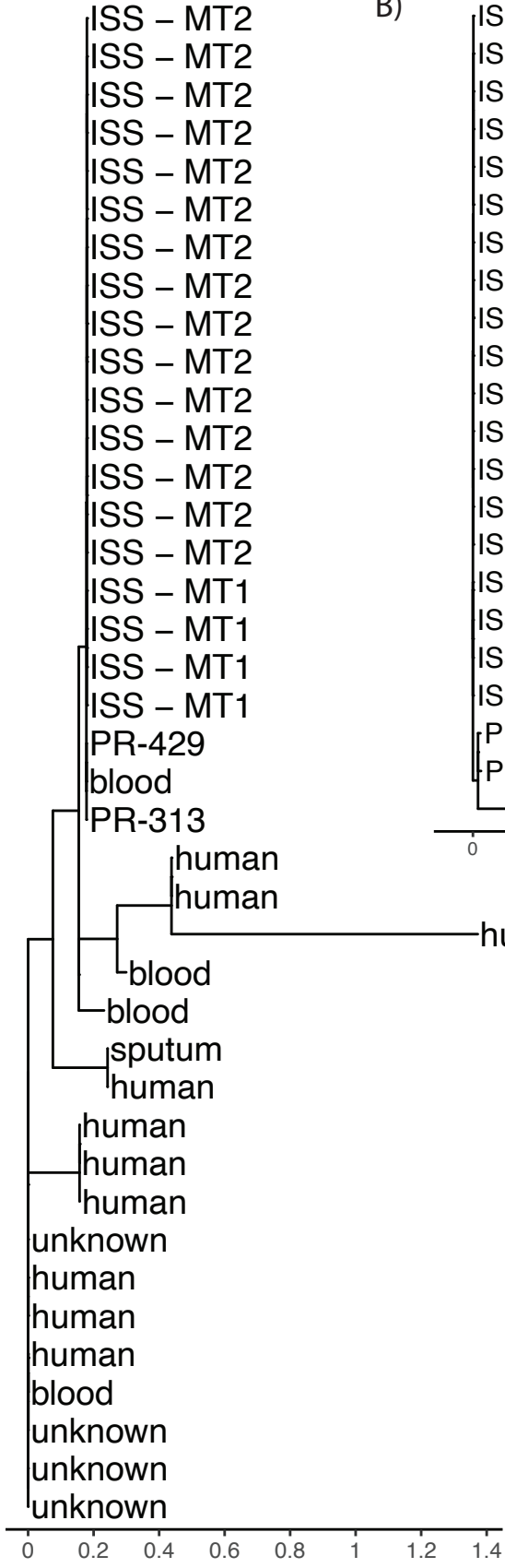

B)

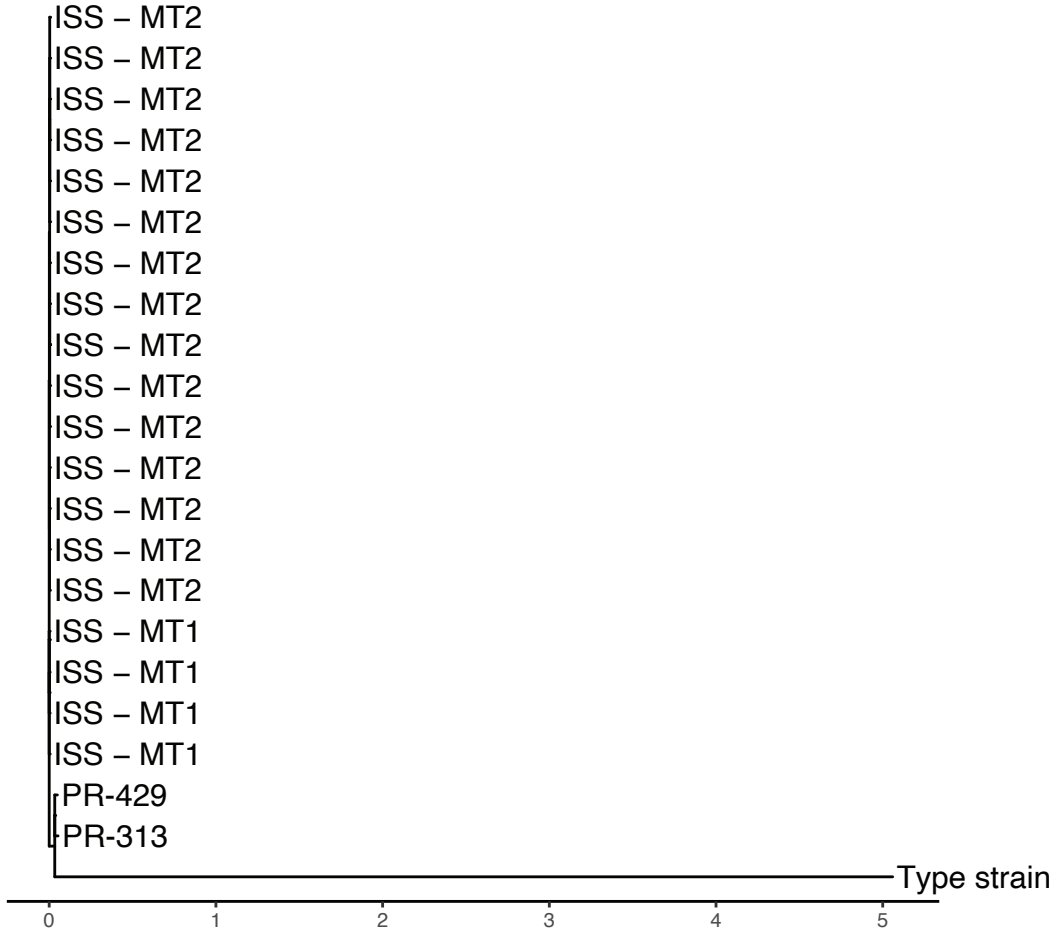

Supplement: Supplementary file 5 — Additional file 4: Supplementary Figure 1. Two alternate phylogenetic trees built with different SNP-calling pipelines, A) SPANDx and B) Snippy. The former contains the genomes most closely related to the ISS A. pittii isolates for which we were able to access raw reads. The latter was generated with raw reads for the ISS isolates and the two closely related Earth strains that were used in antibiotic testing. [file 40168_2022_1358_MOESM4_ESM.pdf]

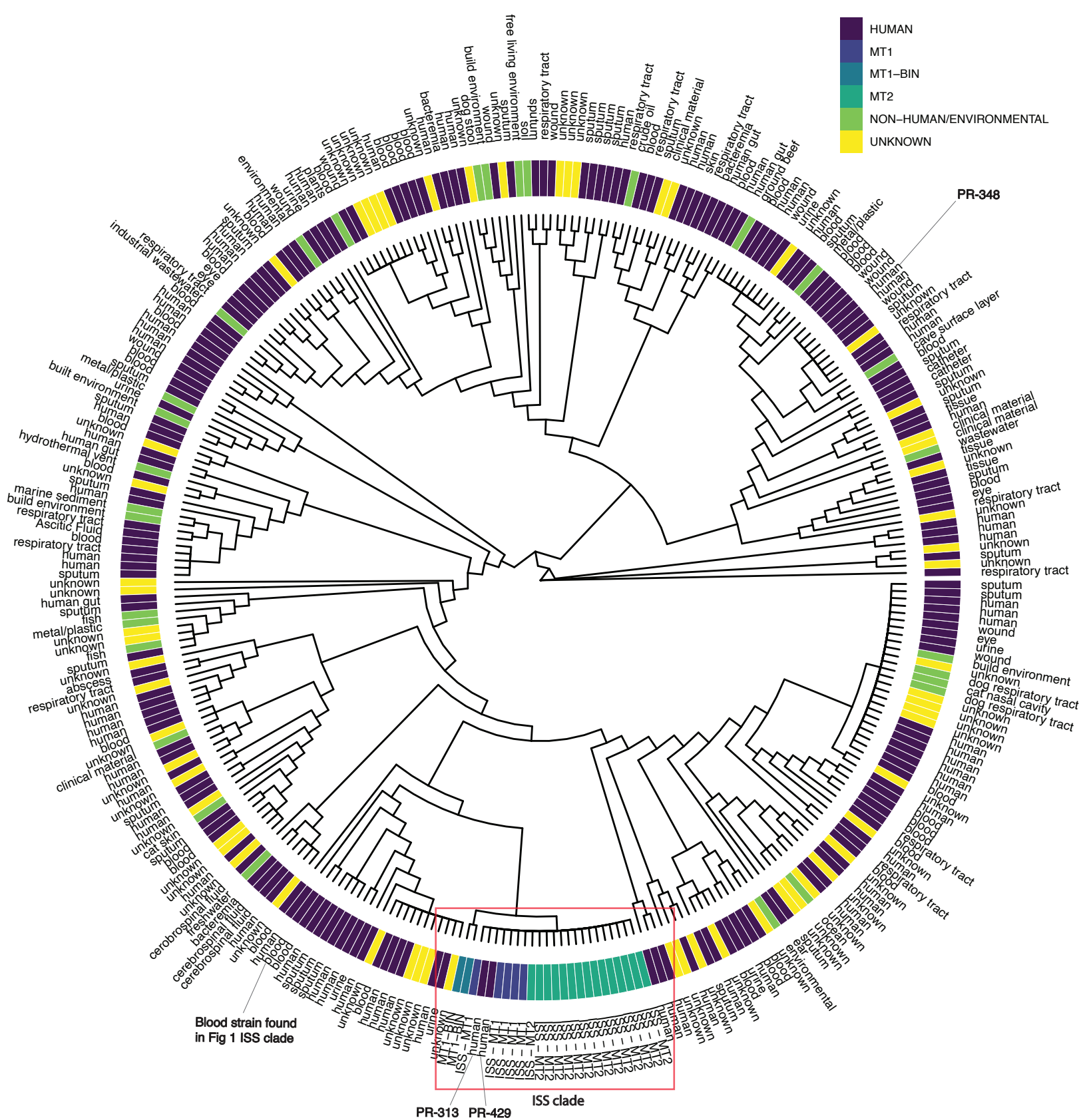

Supplement: Supplementary file 6 — Additional file 5: Supplementary Figure 2. A cladogram from GTDB-tK taxonomic annotations comparing the A. pittii strains from earth versus those identified on the International Space Station. We label the clade containing ISS-associated microbes as the “ISS clade”. We additionally indicate the position of the three clinical isolates that underwent antimicrobial resistance screening as reported in Supplementary Table 2. The labeled “blood strain” lies in the ISS clade in Fig. 1. [file 40168_2022_1358_MOESM5_ESM.pdf]

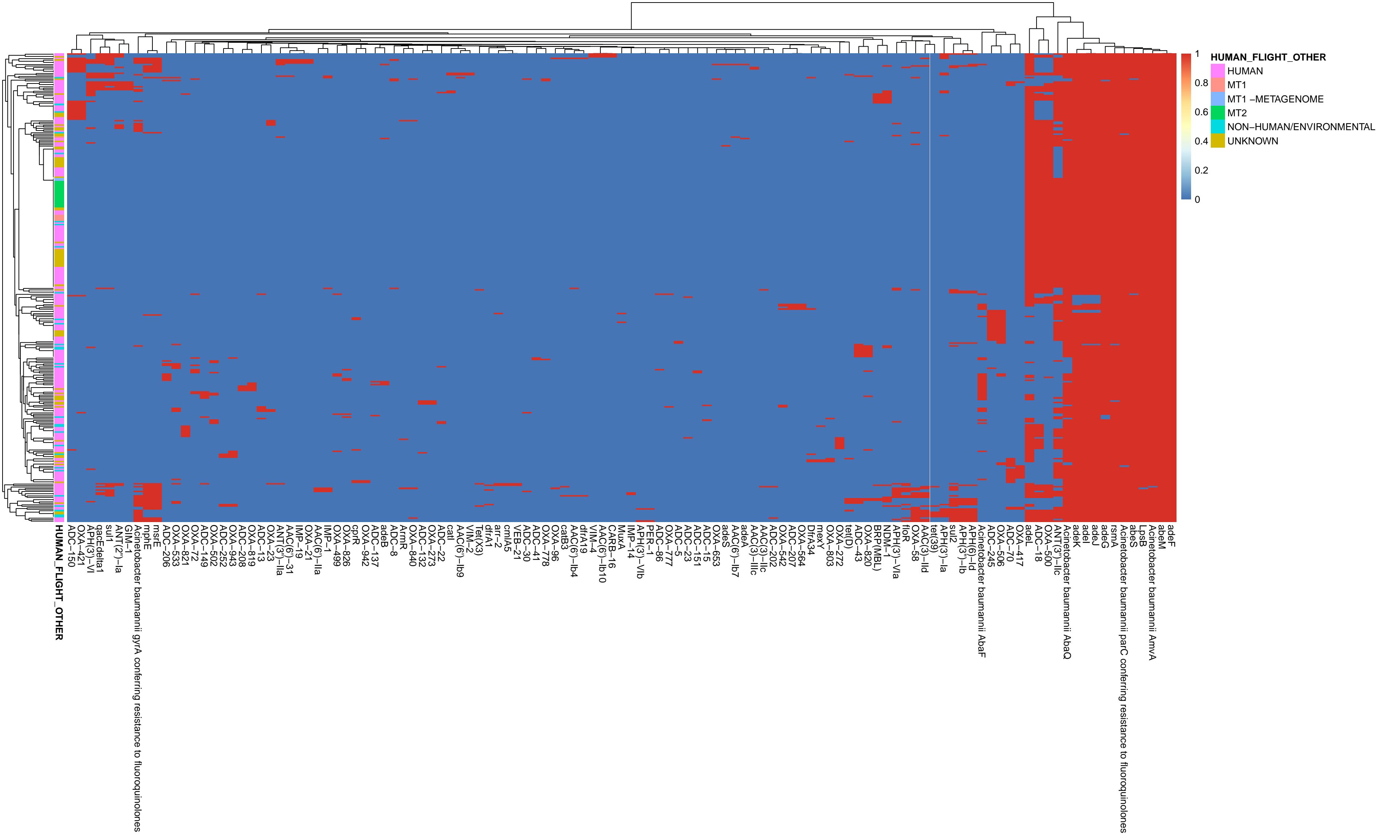

Supplement: Supplementary file 7 — Additional file 6: Supplementary Figure 3. The presence of Antimicrobial Resistance Ontologies (AROs) across all genomes. [file 40168_2022_1358_MOESM6_ESM.pdf]
